# Education as a dimension of human development: A Provincial-level Education Index for Ecuador

**DOI:** 10.1371/journal.pone.0270932

**Published:** 2022-07-08

**Authors:** Marta Guijarro-Garvi, Belén Miranda-Escolar, Yira Tatiana Cedeño-Menéndez, Pedro Benito Moyano-Pesquera

**Affiliations:** 1 Department of Economics, University of Cantabria, Santander, Spain; 2 Economics and Public Policy Research Group, University of Valladolid, Valladolid, Spain; 3 Department of Applied Economics, University of Valladolid, Valladolid, Spain; 4 Faculty of Economics, University Eloy Alfaro of Manabí, Manta, Ecuador; University of Milano–Bicocca: Universita degli Studi di Milano-Bicocca, ITALY

## Abstract

This article deepens in the differences in educational development between the Ecuadorian provinces and in their evolution over time by estimating the Provincial-level Education Index. This index is built using the micro-databases of the two latest rounds of the Ecuador`s Living Standards Measurement Survey (2005–2006 and 2013–2014). The results show an overall increase in the educational development of the Ecuadorian provinces, as well as a slight reduction in inequality. However, differences between them continue to exist. Underlying our results, which are consistent with the provincial production structure and socioeconomic context, some public policies seem to affect the educational sector, as their impact has been evidenced during the period covered in this research.

## Introduction

In its first report on the Human Development Index, published in 1990, the United Nations Development Programme (UNDP) considered health, education, and income as the three key dimensions of human development. Also, the Human Development Index (HDI) as a “more genuine measure of socioeconomic progress” [1] was presented in said report. As does the human capital approach [2–7], the human development approach considers that education enhances the capital incorporated into the production process. In addition, educated individuals obtain a greater monetary return, since, due to the close link between real capital and the formation of human capital, education increases productivity [8]. Generally, more years of schooling leads better paid jobs, lower risk of material deprivation, and higher levels of subjective well-being [9].

Furthermore, education can be related to health through three interacting pathways: acquired health knowledge and its application in daily life; employment and income, and social and psychological factors, including sense of control and social status [10]. In general, people that have received more years of education have longer life expectancy and better health, as they adopt healthier lifestyles −such as regular physical exercise, refraining from smoking, and undergoing regular medical check-ups [11, 12].

Education is also a right which was established in 1948 by the United Nations Universal Declaration of Human Rights [13]. Moreover, it is a key factor for improving people’s quality of life. In addition, amongst other targets, the fourth Sustainable Development Goal (SDG) seeks to ensure that every child completes free primary and secondary education by the year 2030. This goal also aims to provide equal access to attainable technical training, eliminating gender or income-related disparities, and achieving universal access to quality higher education. Furthermore, education is behind the accomplishment of many other SDG, such as eradicating poverty–given that one of its many expressions concerns the difficulty of access to basic services such as education–and reducing inequality through greater investment in education [14]. As a result, education is widely recognized as an instrument which can help people to overcome poverty [15], improve their well-being and curb their dependence on social protection programs [16–18]. Income and work status depend on the level of schooling achieved, so breaking the continuing income inequality is essential to reduce inequality in education. It is therefore necessary to promote more equal access to the quantity and quality of education [7].

The *Buen Vivir* (Good Living) approach is grounded on the paradigm of human development [19]. Indeed, it goes further by placing collective well-being above individual well-being (see, e.g., [20]). Most of its many meanings agree that the origin of *Buen Vivir* lies in the culture of the indigenous peoples and that equality and environmental sustainability are core principles [21]. *Buen Vivir*–or *sumak kawsay* (in *kichwa*)–is a multidimensional concept [22] enshrined in the 2008 Ecuadorian Constitution [23], with equity being one of its principles [19]. The Ecuadorian policies of *Buen Vivir* are reflected in the *Planes Nacionales de Desarrollo* (National Plans for Good Living), which are the principal mechanisms of national political management [24–26]. These policies are geared towards bolstering individuals’ capabilities and potential by considering access to education as one of the central dimensions of well-being [27, 28].

The values of the Human Development Index (HDI) in Ecuador and, more specifically, those related to its education index increased between the years of the study (from 0.600 in 2006 to 0.695 in 2014) [29]. Thereby, they evidenced an improvement in educational well-being in Ecuador over the period analyzed. However, both the HDI and its dimension indices are national averages and therefore mask inequalities in the level of well-being within a country [1]. Interterritorial inequalities are related to factors that heighten the inequalities between individuals or social groups, and they might also be linked to social conflicts. This is especially true in the presence of weak political regimes [30], with the consequent deterioration of social cohesion [31]. This justifies the exploration of such inequalities at the subnational level [32]. Our work is in the line of research adopted by works that have estimated HDI values for different population subgroups [33–38]. Furthermore, understanding these differences will serve as a starting point for the achievement of SDG 10, i.e. the reduction of inequality within and between countries.

In Ecuador, the “horizontal” inequalities between groups that have been established for cultural reasons [39] only exacerbate the risk of poverty and social exclusion. This is particularly remarkable amongst the indigenous and Afro-descendant populations [40–42], as well as between the different areas of the country. Ecuador has four regions, three of which are continental (Costa, Sierra, and Amazonia). In contrast, the fourth region, the island region of the Galapagos archipelago, is on the Pacific Ocean. In turn, the regions are divided into provinces, which are the first-level political-administrative units. The geographical location of the population may stifle the academic achievement of children and youngsters, leading them to fall behind or leave school early, since not all the provinces are able to offer the same educational quality (poorly qualified teachers) or adequate school infrastructures (difficulties regarding access, electricity, drinking water or sanitation). Moreover, the situation of child labor in those provinces whose productive specialization and the nature of the local labor market increase the opportunity costs of education must be considered. Finally, ethnic and linguistic fragmentation–which is unequally distributed throughout the various provinces–is a major challenge for the Ecuadorian education system, as it is results in a need to take account of the idiosyncrasy of children in the different areas of the country and to guarantee education in indigenous languages, as a first language, and in Spanish, as the language of intercultural relations [43, 44].

Given that one of the priorities of *Buen Vivir* is the reduction of inter-territorial inequalities [45], the analysis of these inequalities will provide a key tool for decision-making [46]. Thus, this paper provides information on the educational dimension of human development in the Ecuadorian provinces and its evolution over the period covered by the last two rounds (2005–2006 and 2013–2014) of the Ecuadorian *Encuesta de Condiciones de Vida* (Living Standards Measurement Survey, or LSMS). For this purpose, a Provincial-level Education Index (PEI) is calculated by using the micro-databases from these two latest rounds of the LSMS. The Ecuadorian LSMS is a household survey that offers a multidimensional concept of individual well-being which is in line with the human development paradigm underlying the HDI. However, data provided by a LSMS must be converted into information [47]. In the context of this research, measuring a country’s educational development requires the interpretation of data based on its educational reality. Thus, to carry out this measure, we must consider the structure of the national education system of Ecuador in its latest reforms, focusing on the period covered in the two rounds of the LSMS. Furthermore, for a correct interpretation of provincial differences, we need to explore our understanding of Ecuador’s educational reality within the territorial context of the country.

To obtain the PEI, we first estimate the variables mean years of schooling and expected years of schooling for each Ecuadorian province. Secondly, we combine these indicators following the UNDP method for obtaining the education component of the HDI. The result is a comparable index that provides insight into the level of educational development of Ecuadorian provinces. In particular, the PEI allows us to situate this level of educational development in the context of Latin American and Caribbean (LAC) countries with an upper-middle income level such as Ecuador. Finally, we estimate the level of Ecuadorian educational inequality in 2006 and 2014, as well as the contribution of each component of the PEI and of each Ecuadorian province to this inequality.

Although our results indicate an increase in the educational development of Ecuadorian provinces and a slight reduction in inequality, there are still differences between provinces. Our findings are consistent with the provincial socio-economic context and show the effects of certain education policies.

Given that the methodological structure of the Ecuadorian LSMS has remained unchanged since its first round, it is to be hoped that the procedure put forward to estimate the PEI might be replicated in future rounds. Thereby it will enable us to extend current knowledge on the distribution of education in Ecuador at a provincial level and thus help public authorities to take more effective education policy decisions. It is worth noting that LAC countries conduct continuous household surveys that provide information on different aspects of the country’s well-being and some of them conduct LSMS on a continuous basis. Therefore, the present research could be used as a guide for obtaining educational development indices at the subnational level in other LAC countries.

## 2. Ecuador in the context of Latin American and Caribbean education policies

Most of the education policy reforms that have been implemented in LAC countries in recent decades have revolved around three axes [48–51]: (i) institutional reforms aimed at causing changes in the management and functioning of the education system, promoting decentralization in decision-making, granting more autonomy to schools, and encouraging local and family participation in educational activities; (ii) improvements in quality and equity,–mainly in schools and social sectors with lower incomes and academic performance–, through free textbooks, school materials and uniforms, investments in school infrastructure and teaching materials, and improvements in teacher qualification and training, and (iii) new funding sources aimed at improving the efficiency and effectiveness of public spending on education: education vouchers, special funds for the allocation of resources based on academic achievement and/or the number of students enrolled, and grants for children coming from vulnerable families with scarce socioeconomic resources.

Despite the efforts made, diagnoses of the educational situation in the region show that there are still problems that need to be solved in terms of quality, equity, and efficiency. Although decentralization has improved educational coverage and allowed greater participation of parents and local communities in the functioning of school life, learning outcomes are much less encouraging [48, 50, 52]. These shortcomings may lie behind the absence of accountability policies and standards-based reforms, which introduce elements that have traditionally existed in other education systems–such as school inspection and external evaluation of learning outcomes, from which guidelines for school improvement can be derived–, especially in Europe and North America [53].

Although these processes have been in place for years in some LAC countries, such as Colombia and Chile [54, 55], the lack of reliable information and appropriate indicators has limited their widespread incorporation. To this should be added the consequences associated with the procedures that aim to achieve higher quality in teaching by assigning rewards and sanctions based on individual performance. However, given the large differences in educational resources and in student composition (different ethnicities) between schools (rural vs. urban), as well as the complex nature of the educational process itself, it is very difficult to determine the net effect in each case. Therefore, rewards and sanctions can often be unfair, affecting not only the equity but also the productivity of the mechanism itself [56]. Nevertheless, many of the education reforms that have been implemented in the region in recent years have moved in this direction.

Ecuador’s educational problems are no different from those of other LAC countries, with high repetition and dropout rates as two characteristics of the education systems in the region [56, 57]. Also, poor academic achievement should be added to these phenomena [40–42].

These problems were behind the successive educational reforms implemented by Ecuador in recent decades. Among them, the 1983 *Ley Orgánica de Educación* (Education Act, or LOE) [58] should be highlighted. This Act, together with the *Reglamento General de la Ley Orgánica de Educación* (General Rules of the Education Act) in 1985 [59], set out the structure of the non-university education (S1 Table).

In the belief that education is one of the most effective means of preventing hereditary poverty, the Ecuadorian government designed and promoted the first *Plan Decenal de Educación* (Ten-Year Education Plan) 2006–2015. The reforms stemming from the Ten-Year Education Plan were first conducted in 2009, modifying the levels of general basic education and, subsequently in 2011, with the first curricular reform of the general unified Baccalaureate. These reforms were consolidated through the approval of the *Ley Orgánica de Educación Intercultural Bilingüe* (Intercultural Bilingual Education Act, or LOEI) in 2011 [60]–last amended in 2021 [61]–which is currently in force. Schooled education consists of three levels: Initial Education, General Basic Education, and Baccalaureate (S2 Table). The major changes which the LOEI brought into non-university education were that Primary Education (six years) and Lower Secondary (three years), from the previous system, became the ten-year General Basic Education by also including one further year, corresponding to Basic Preparatory Education. This latter educational level ceased to be optional after the approval of the LOEI and, in turn, Diversified Secondary Education (three years) came to be known as Baccalaureate (S1 and S2 Tables).

In the field of university education, reforms were set out in the *Leyes Orgánicas de Educación Superior* (Higher Education Acts, or LOES) of 2000 and 2010 [62, 63]. More recently, in 2018,–although outside the period analyzed in this paper–the *Ley Orgánica Reformatoria a la Ley Orgánica de Educación Superior* (Reform Act) was approved [64]. These reforms have mainly affected institutions that conform the national higher education system, in an effort to bolster it and ensure equal opportunities for students. Moreover, the duration of studies was also extended, now ranging from two years for full-time students in the High-level Technician Degree (increasing the number of teaching hours from 1,950 to 3,200) to six years for the Degree in Human Medicine. Postgraduate studies (Master’s Degrees) have a minimum duration of one and a half years (three semesters or other periods equivalent to 48 weeks) and they may entitle admission to a doctoral program. The PhD programs lasts a minimum of six semesters and a maximum of fourteen, plus two grace periods.

Along with these reforms, other policies that may have contributed to the improvement of Ecuador’s educational development, such as the *Bono de Desarrollo Humano* (BDH), should be considered. The BDH is a conditional cash transfer (CCT) program whose origin lies in the *Bono Solidario* of 1998 [65, 66]. CCT programs were implemented by LAC countries at the request of international organizations between the end of the past century and the start of the 21^st^ century. One of its main tools was the payment of a cash transfer conditional upon universal access to social services in health and education [67, 68]. These programs, which were key symbols in poverty reduction [69], consider education as a vital factor in human development [70]. The BDH is part of the poverty eradication policy foreseen in the National Plans for Good Living, acting in close collaboration with the Ten-Year Education Plans.

In fact, the BDH’s objectives included fostering school reinsertion and ensuring continued school attendance of children and adolescents [71]. Initially, co-responsibility in education was applied to selected families with children between the ages of 6 and 16. Throughout its over twenty years of existence, the BDH has undergone several changes which have affected, among other things, criteria for selecting beneficiaries and the amount of the transfer. The latest reform of the BDH in 2019 adds to the fixed amount of USD 50 per month transferred to the heads of beneficiary households in extreme poverty, an additional variable component depending on the number and age of children under 18 years old. In this vein, the maximum amount that the monthly cash transfer can reach by adding the fixed and variable components is USD 150 [72]. This is the line followed by some studies that consider that a differentiated cash transfer according to the level of poverty (higher for the poorest), the age of the children (lower for the youngest) and the ethnic group (higher for indigenous and Afro-Ecuadorian children) is essential [41].

## Data and methods

### Data: The Ecuadorian Living Standards Measurement Survey

Since the 1990s, there has been a considerable increase in the availability and robustness of national household surveys in LAC countries. Moreover, the subnational nature of many of the policies implemented in the countries of the region has forced surveys to be representative at increasingly higher levels of disaggregation [73]. Among these surveys, LSMSs are household consumption and expenditure surveys which contain sections on a wide number of social topics, such as education, nutrition, health, or fertility (for a classification of household surveys, see e.g. [74]). Although LSMSs are best suited for inequality analysis [75], the fact that these surveys are more complex and costly than other household surveys means that LSMSs are only occasionally conducted in LAC countries.

Initially, LSMSs formed part of the Living Standards Measurement Study, funded by the World Bank [76]. This study tool offered a holistic vision of well-being, focusing attention on its conditioning factors and including those derived from the implementation of government policies [77]. After 1996, LSMSs came to form part of the Program for the Improvement of Surveys and Measurement of Living Conditions in LAC, as a joint initiative of the Inter-American Development Bank, the World Bank, and the Economic Commission for Latin America and the Caribbean (ECLAC) (for details of countries that participated in this program and their surveys, see, e.g. [75]). This program continues to support countries in conducting household surveys, focusing on improving the knowledge and application of household survey methodologies [47].

In Ecuador, the first round of the LSMS was conducted by the *Servicio de Capacitación Profesional* (Professional Training Service), whilst the following rounds were implemented by the *Instituto Nacional de Estadística y Censos* (National Department of Statistics and Census, or INEC). Since its first round in 1994, the LSMS was designed to gain an insight into the distribution of the population’s well-being and to glean information on the impact of the measures of macroeconomic adjustment and government programs aimed at reducing poverty. In fact, the general objectives of the Ecuadorian LSMS are aligned with the logic of *Buen Vivir*, providing the necessary tools to implement policies that contribute to the elimination of population inequalities. Furthermore, due to its methodological design, the LSMS is a fundamental statistical instrument in the study of the living conditions of the Ecuadorian population [78].

As the aim was to also obtain an updated baseline to measure compliance with the United Nations Millennium Development Goals [79], the LSMS provide information concerning the different aspects and dimensions of well-being in Ecuadorian households, which constitute the units of information (the methodology used in this survey may be seen in [78]). The multidimensional concept of individual well-being offered by the Ecuadorian LSMS in line with the human development concept of the HDI and its intermediate indexes makes it the most suitable household survey for the study of educational development in the Ecuadorian provinces. To measure Ecuadorians’ quality of life, the variables considered in the LSMS are classified into sections. In particular, the section devoted to the educational dimension of human development aims to ascertain the level of literacy among the population, the level of non-attendance or household spending on education, among other aspects.

The micro-databases of the fifth round (2005–2006) and the sixth round (2013–2014) of the Ecuadorian LSMS used in this paper contain information on 55,666 and 109,694 individuals, respectively [80, 81]. The two rounds of the LSMS adopt a similar methodology in terms of the structure of the questionnaire, the variables used, and the treatment given to the information by the executing entity, which endows the results obtained with a high degree of reliability. Both surveys offer national, regional, and provincial representativeness [78, 82]. Their methodology is based on the expansion factor of each individual surveyed, defined as the inverse of the probability of choosing a household and its members in each province [78, 82]. This method allows the indicators derived from their use to be generalized to the population represented in the survey [78] and avoids the problem of missing values in certain household surveys (see, for example, [83]).

### Educational development in Ecuador’s provinces: The Provincial-level Education Index and its components

Our use of the micro-databases of the two rounds of the Ecuadorian LSMS was established on a selection of identification and education variables of the population surveyed. This enabled us to estimate the indicators that make up the PEI for Ecuadorian provinces, following the method for the education index of the HDI applied by the UNDP since 2010. These indicators, which respectively reflect a population’s levels of both current and future education, are mean years of schooling (MY) and expected years of schooling (EY) [84]. Based on the UNESCO definitions [85], both indicators are felt to be of interest by the INEC within the educational dimension of well-being [86].

Thus, for each province, we obtained the MY indicator as an average of school years studied by the population aged 25 or over:

$$\text{MY}=\sum_{j}\frac{j\cdot n_{j}}{N}$$

where *n*_*j*_ is the number of individuals with *j* years of education out of a total of *N* individuals aged 25 and over.

Undoubtedly, this indicator was the most difficult to estimate given that different education systems have existed (still exist) in Ecuador at the same time, due to the enactment, withdrawal and, on occasions, intertwining of successive reforms. It should therefore be remembered that those people surveyed might have gone through one education system, another, or indeed both. This complex scenario is further compounded by the fact that neither of the two rounds of the LSMS offers a direct question addressing the number of years an individual has spent in the education system, forcing us to merge various answers from each respondent to obtain the required information.

S3 and S4 Tables sum up the main aspects of the procedure undertaken to estimate the mean years of schooling indicator for each province using the fifth and sixth round of the LSMS, respectively. The first column of each table specifies the possible answers to the question in the questionnaire concerning the “highest level of education attended or completed” which, together with the “highest year of education passed” (second column of both tables), provided us with a base from which to obtain the number of years of schooling of the respondent. The third column sets out the maximum number of years an individual attended school, according to the level of education indicated in their answer to the previous questions. The last column contains the rest of the questions used, in addition to some of the technical issues we considered when interpreting certain answers. Finally, to estimate the mean years of schooling indicator, we multiplied the number of years of schooling of each individual by the expansion factor.

To estimate the EY indicator for each province, we followed the definition proposed by the INEC to calculate this indicator at a national scale [78, 82]. To do this, we added the attendance (or enrolment) rates for each age between 5 and 17, together with the corresponding gross attendance (or enrolment) rate for the 18–22 aged group multiplied by five:

$$\text{EY}=\sum_{j=5}^{17}\frac{u_{j}}{v_{j}}+\frac{\sum_{j=18}^{22}u_{j}}{\sum_{j=18}^{22}v_{j}}\cdot 5$$

where *u*_*j*_ is the number of individuals of age *j* enrolled out of a total of *v*_*j*_ individuals. Finally, we applied the expansion factor to each individual enrolled in each age group.

We estimated the PEI for the provinces of the regions: Sierra (Azuay, Bolívar, Cañar, Carchi, Cotopaxi, Chimborazo, Imbabura, Loja, Pichincha, and Tungurahua); Costa (El Oro, Esmeraldas, Guayas, Los Ríos and Manabí), and Amazonia (Morona Santiago, Napo, Pastaza, Zamora Chinchipe, Sucumbíos and Orellana). We did not take into consideration the single-province region of Galapagos due to a lack of representativeness in both LSMS [78, 82]. Although the fifth round of the LSMS is only representative at the regional level for Amazonia, we estimated the PEI for its provinces for this round as well. Sometimes it is not possible to find representative samples of the population, which raises the dilemma between the desire to obtain more accurate results–whose reliability could be statistically evaluable–, and the practice of calculating less accurate results [87]–which allow us to approach the reality to be analyzed–. In this study, we critically evaluated this particular context [88] and opted for the second option. Therefore, we were cautious in interpreting the results for these Amazonian provinces when they referred to the fifth round of the survey.

Processing the information at a provincial level involved identifying the codes of the country’s territorial divisions. This was by no means a trouble-free procedure, as in 2007 the province of Santo Domingo de los Tsachilas (hereafter Santo Domingo) and in 2008 the province of Santa Elena were created, which did not exist in the fifth round of the LSMS, and whose populations belonged to Pichincha and Guayas, respectively [89]. The creation of new political-administrative units affects the construction of chronological series of socioeconomic indicators for the various spatial levels. These new units, which tend to be established based on a grouping of smaller units, as is the case here, change the previously existing boundaries and make spatial standardization of the time series even more difficult. Consequently, with a view to drawing comparisons between the PEI values for 2006 and 2014, it was necessary to calculate a “combined” value of the estimations for each of the education indicators. These estimations were made using the micro-databases from the sixth round for Pichincha and Santo Domingo, on the one hand, and for Guayas and Santa Elena, on the other. In order to obtain these values, we used the means estimation procedure in the stratified sampling, taking the initial estimations of the values of indicators in the four provinces and using, for this case, the weightings determined by the corresponding population ratios obtained from the 2014 population forecasts [90].

To validate our results, we compared our provincial estimations of the mean years of schooling indicator, in 2014, to the information offered by the *Secretaría Nacional de Planificación y Desarrollo* (National Department of Planning and Development, or SNPD) [91] on this variable at a provincial level for said year, which was also obtained from the microdata of the sixth round of the LSMS. This is the only information from official statistics for Ecuador that may be deemed “homogeneous” *vis-à-vis* drawing comparisons. In any case, the mean years of schooling provided by the SNPD was estimated on the population aged 24 and over, which does not allow comparisons with UNDP data, unlike the methodology proposed in this paper. Given that the estimations were conducted on different population groups, we used the Spearman´s rank correlation coefficient to compare the classifications determined on the provinces both by our estimations and by those from the SNPD in order to assess the validity of our results. Moreover, and bearing in mind that the SNPD statistics offer data for Santo Domingo and Santa Elena, we included the estimations of the indicator for these provinces in said analysis. The estimated value of the coefficient equal to 0.99 and statistically significant at the 1% level is indicative of near perfect concordance between the classifications given by the two indicators and therefore evidences the consistency of our estimations. Furthermore, there are no official statistics for the expected years of schooling indicator at a provincial level in Ecuador.

Having estimated the education indicators for each province, we transformed both indicators into normalized indices between 0 to 1 by the UNDP goalposts used since the 2014 Human Development Report: minimum of 0 years for both indicators, and maximum of 15 and 18 years for mean years of schooling and expected years of schooling, respectively [92]:

$${\text{MYS}}_{i}=\frac{{\text{MY}}_{i}-0}{15-0}$$

$${\text{EYS}}_{i}=\frac{{\text{EY}}_{i}-0}{18-0}$$

where MYS_*i*_ and EYS_*i*_ are the mean years of schooling and the expected years of schooling normalized indices for province *i*, respectively. For each province, *i*, we calculated its PEI_*i*_ as the arithmetic mean of the normalized education components (i.e., (MYS_*i*_ + EYS_*i*_) / 2). This is the methodology used by UNDP to obtain the education index of the HDI since 2014 [92]. PEI_*i*_ ranges from 0 (minimum level of educational development) to 1 (maximum level of educational development). Applying the same method of estimating the PEI that the UNDP uses to construct the educational component of the HDI allowed us to “situate” the level of educational development of Ecuadorian provinces in the international context. More specifically, it enabled us to compare Ecuador`s educational well-being to that of LAC countries with similar socioeconomic characteristics.

In this sense, to illustrate the spatial distribution of Ecuadorian educational development, we took as a reference the classification by quartiles corresponding to the PEI of Ecuadorian provinces in 2014. We used the quartile ranking because it is the procedure adopted by UNDP to rank countries according to their level of human development based on their HDI values (low, medium, high, and very high) [93]. It should be mentioned that the HDI is meant to order geographic units, allowing comparisons between countries and between different time periods. Therefore, what is important is not so much the value of the HDI in a given country, but its rank or order number in relation to the rest of the countries. This is equally valid for each of the intermediate indexes that compose the HDI, such as the education index.

To complete the above information, we determined the level of educational development of each province compared to that of the countries with available information for 2014 in the UNDP database [29] Thus, following the aforementioned UNDP methodology for its HDI, we calculated the first, second and third quartiles, respectively, of the HDI education index distribution for 2014. These quartiles are the thresholds that allowed each province to be classified according to its level of educational development.

### Statistical analysis of Ecuadorian educational inequality across provinces

We explored whether the provinces with the lowest educational development in 2006 were the ones that grew the most between 2006 and 2014. To see whether this convergence process occurred, we estimated the Spearman`s rank correlation coefficient, *ρ*, between the ranking of the provinces determined by their PEI in 2006 and that given by the annual mean variation of the PEI between 2006 and 2014 [94, 95], and we replicated this analysis for the two components of the PEI. To analyze the significance of the results we used the statistic $t=\rho \cdot \sqrt{(N-2)/\left(1-\rho^{2}\right)}$, where *N* is the number of provinces; assuming no concordance between rankings, this statistic follows a *t*-distribution with *N*—2 degrees of freedom.

To quantify the inequality in Ecuador’s educational development across provinces and to gain an insight into its evolution over the period analyzed and the contribution of each component of the PEI makes to overall educational inequality, we used the square of the coefficient of variation, CV^2^. This choice is due to the simplicity of this measure in terms of calculation and interpretation and because it offers the desired properties of a measure of inequality (for a review of measures of inequality and their properties, see e.g. [96]), one of which is the possibility of its additive decomposability by factors [97].

As is well known, the square of the coefficient of variation of PEI, ${\text{CV}}_{\text{PEI}}^{\text{2}}$, is equal to $\sigma_{\text{PEI}}^{2}/\mu_{\text{PEI}}^{2}$ where $\sigma_{\text{PEI}}^{2}$ and $\mu_{\text{PEI}}^{2}$ are, respectively, the variance and the square of the mean of the PEI. The closer the value of ${\text{CV}}_{\text{PEI}}^{\text{2}}$ is to zero, the lower the educational development inequality. Conversely, since this coefficient is not upper bounded, the larger its value, the greater the inequality.

Considering that PEI = (MYS + EYS) / 2 and following Shorroks’s methodology [97], the “natural” decomposition of the square of the coefficient of variation is

$${\text{CV}}_{\text{PEI}}^{\text{2}}=\frac{1}{4\cdot \mu_{\text{PEI}}^{2}}\left(\sigma_{\text{MYS}}^{2}+\sigma_{\text{MYS},\text{EYS}}\right)+\frac{1}{4\cdot \mu_{\text{PEI}}^{2}}(\sigma_{\text{EYS}}^{2}+\sigma_{\text{MYS},\text{EYS}})$$

where $\sigma_{\text{MYS}}^{2}$, $\sigma_{\text{EYS}}^{2}$, and $\sigma_{\text{MYS},\text{EYS}}$ are the variances of MYS and EYS, and the covariate between these education indices, respectively. This decomposition implies that the interaction term, $2\cdot \sigma_{\text{MYS},\text{EYS}}$, is allocated in the same way for both indices. Thus, $(\sigma_{\text{MYS}}^{2}+\sigma_{\text{MYS},\text{EYS}})/4\cdot \sigma_{\text{PEI}}^{2}$ and $(\sigma_{\text{EYS}}^{2}+\sigma_{\text{MYS},\text{EYS}})/4\cdot \sigma_{\text{PEI}}^{2}$ are the proportions of overall educational inequality contributed by MYS and EYS, respectively.

Finally, given that the numerator of ${\text{CV}}_{\text{PEI}}^{\text{2}}$ is the variance of PEI, simple calculations leaded us to see that the contribution of province *i* to overall inequality is equal to ${\left({\text{PEI}}_{i}-\mu_{\text{PEI}}\right)}^{2}/n\cdot \sigma_{\text{PEI}}^{2}$, where *n* is the number of provinces.

## Empirical results

**Fig 1:**
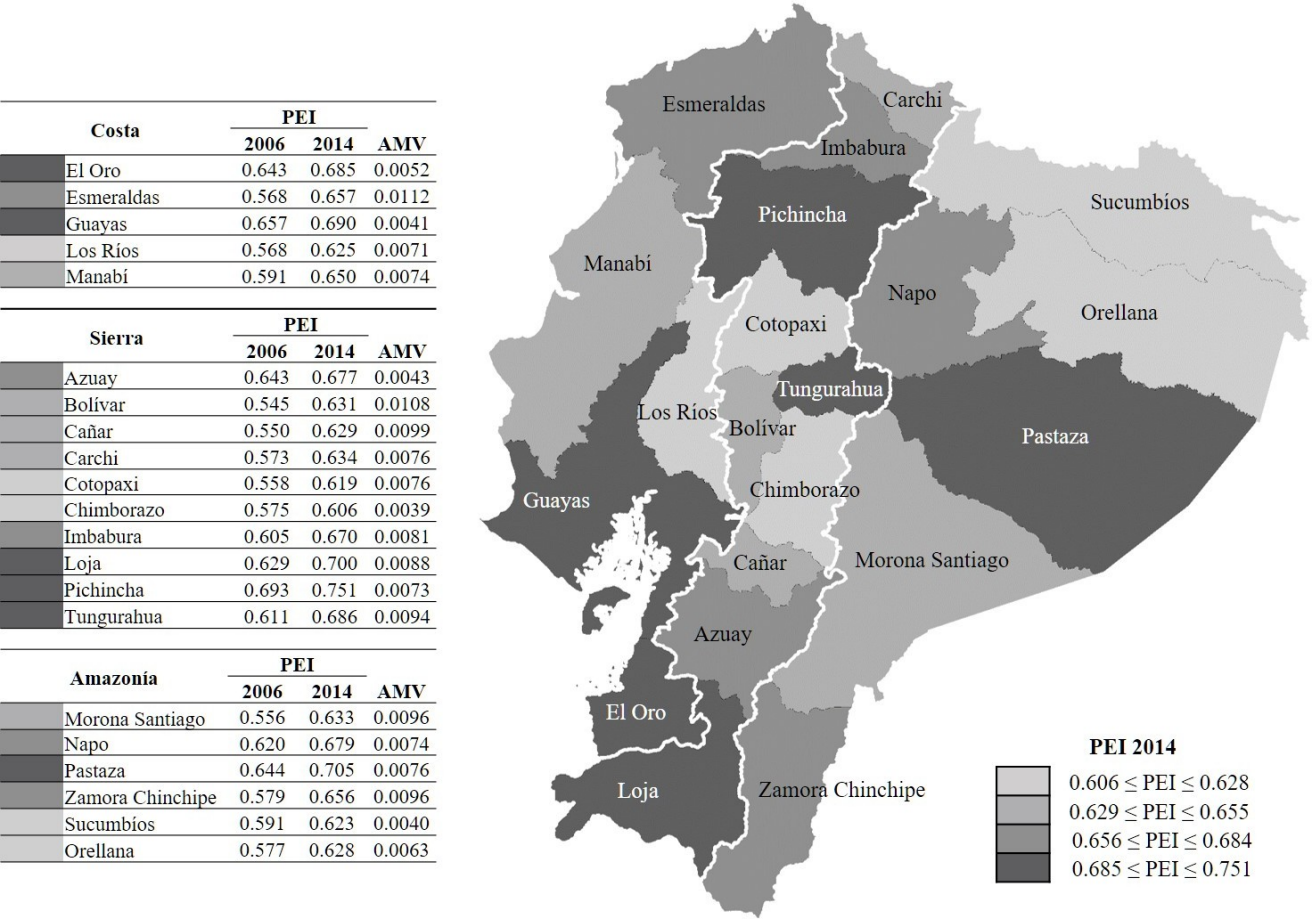
Provincial-level Education Index by quartiles in 2014 and annual mean variation. 2006 and 2014. Notes: the Galapagos region was removed since it was not subject to analysis; the provinces of Santo Domingo and Santa Elena are included in Pichincha and Guayas, respectively; approximate results for the provinces of Amazonia in 2006 due to lack of representativeness in the survey. PEI = Provincial-level education index; AMV = annual mean variation; AMV = (PEI(2014)–PEI(2006))/(2014–2006).

Fig 1 shows the spatial distribution of the PEI in Ecuador for 2006 and 2014 (education indicators data, and PEI and its components data available in S5 and S6 Tables). The Ecuadorian map illustrates the PEI distribution in 2014 taking the classification by quartiles as reference. It can be seen how provinces with a large urban population, such as Pichincha (whose capital is Quito, an eminently metropolitan area), Loja or Guayas, evidenced the highest levels of education, whereas the lowest levels corresponded to provinces with a greater concentration of rural population, such as Chimborazo, Cotopaxi, Los Ríos, and Orellana. Pastaza is an interesting case, as it was the only province in the Amazonia that was among the group of Ecuadorian provinces which displayed the highest level of educational development. Although no regional pattern is evident–since the provinces exhibiting the highest and lowest PEI values were spread around the three regions considered in the study–, what did emerge is that provinces located in the southwest part of the country, such as Guayas, El Oro, and Loja, displayed greater development in education. Meanwhile, Sucumbíos and Orellana–located to the north-east–were two of the provinces lagging furthest behind in the education dimension of well-being.

Table 1 complements the information provided in Fig 1. This table shows the Ecuadorian provinces classified according to their level of educational development in 2014. To conduct this classification, firstly, we considered the values of the education index calculated by UNDP for all countries with available information in 2014 [29]. Secondly, following the aforementioned UNDP methodology for its HDI, we calculated the values 0.500, 0.664 and 0.784, corresponding to the first, second and third quartiles, respectively, of the HDI education index. These quartiles were the thresholds that determined the level of educational development of each province: low, if its PEI was less than 0.500; medium, if its PEI was greater than or equal to 0.500 and less than 0.664; high, if the PEI was greater than or equal to 0.664 and less than 0.784; and, finally, very high, if the PEI was greater than or equal to 0.784. Since all the Ecuadorian provinces had a PEI between 0.606 and 0.751 (see Fig 1), their level of educational development in 2014 was medium or high.

**Table 1:** Ecuadorian provinces according to their level of educational development (2014).

Educational development level	Region	Provinces
Medium ^a^	Costa	Esmeraldas; Los Rios; Manabí
	Sierra	Bolívar; Cañar; Carchi; Cotopaxi; Chimborazo
	Amazonia	Morona Santiago; Zamora Chinchipe; Sucumbios; Orellana
High ^b^	Costa	El Oro; Guayas
	Sierra	Azuay; Imbabura; Loja; Pichincha; Tungurahua
	Amazonia	Napo; Pastaza

Notes: ^a^ Medium: Provincial-level education index greater than or equal to 0.500 and less than 0.664.

^b^ High: Provincial-level Education Index greater than or equal to 0.664 and less than 0.784.

**Fig 2:**
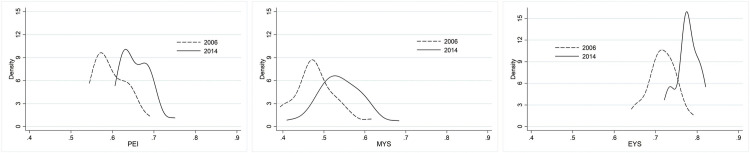
Distribution of the Provincial-level Education Index and its components. Notes: Each line represents the density function of the corresponding distribution. MYS = mean years of schooling index; EYS = expected years of schooling index: PEI = Provincial-level Education Index.

Fig 2 illustrates the dynamics of the PEI distribution and its components, MYS and SYS, for 2006 and 2014, thereby furthering the description provided by Fig 1. All the distributions shifted to the right, with an increase in the mean values of the distributions between the years of the study. This evidences a positive evolution, both in PEI and in MYS and SYS between 2006 and 2014. The figure also shows a slight reduction in the disparity between provinces for the education index and its components between the years in the study, which will be analyzed later. The bimodal nature of the PEI distribution, which was only hinted at in 2006, was more evident in 2014, and reflects the concentration of a group of provinces with an educational level that was above the distribution mean (equal to 0.599, in 2006, and 0.659, in 2014). It is also worth highlighting the different behavior of the distributions of the PEI components. The slight bias to the right of the 2006 MYS distribution virtually disappeared in 2014 and with it so did the number of provinces displaying MYS values below the respective mean value (equal to 0.482, in 2006, and 0.541, in 2014). Regarding the EYS distribution, Fig 2 indicates that most provinces were concentrated around the mean value (equal to 0.715, in 2006, and 0.776, in 2014) for both years of the study. It can also be seen that the density linked to lower values of this indicator tended to decrease in 2014. Despite this, there was a second mode for this indicator in said year, which is related to a group of provinces with below mean values.

**Fig 3:**
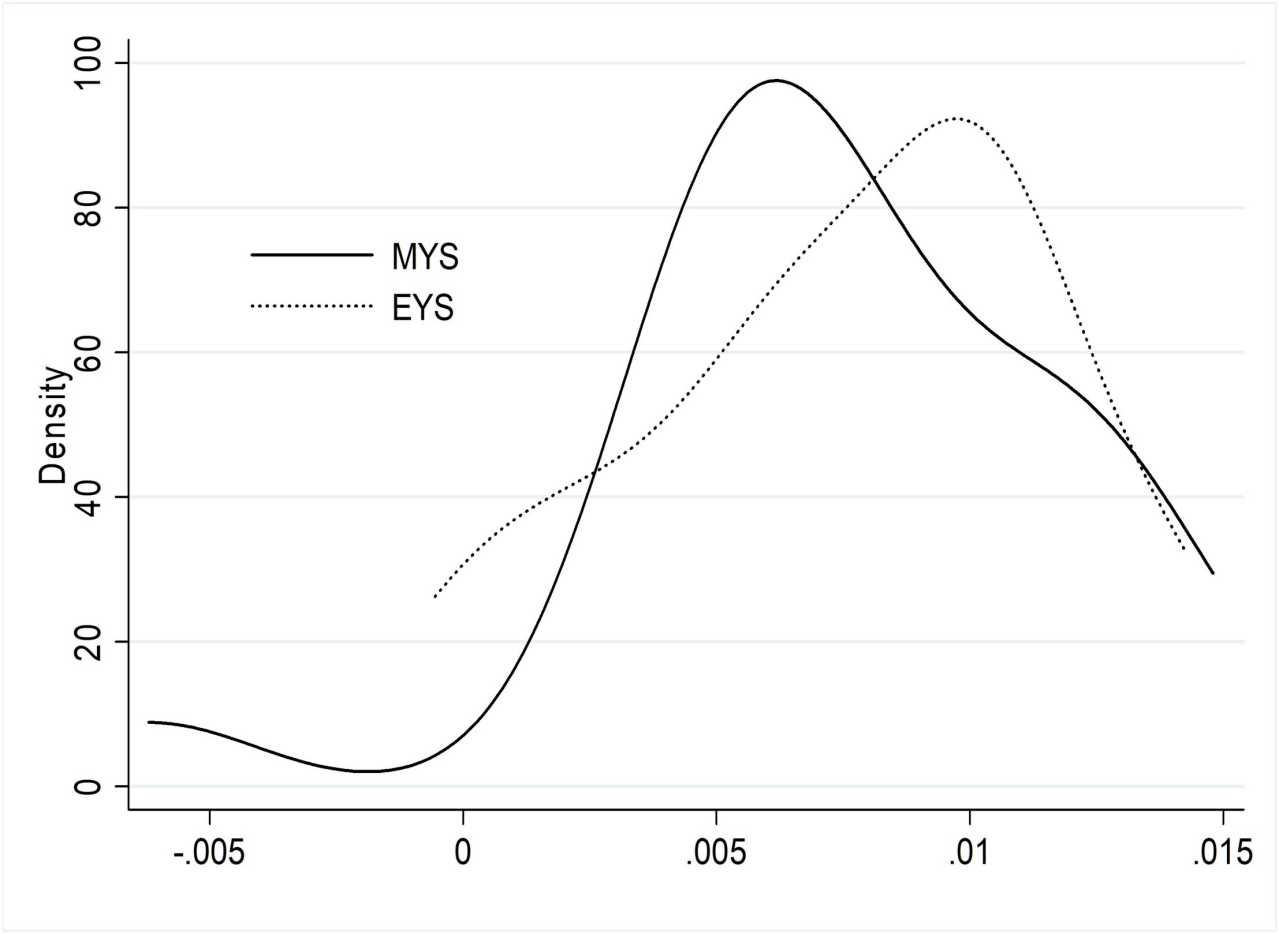
Provincial distribution of the annual mean variation between 2006 and 2014 of the mean years of schooling and the expected years of schooling indices. Notes: Each line represents the density function of the corresponding distribution. MYS = mean years of schooling index; EYS = expected years of schooling index.

The graphs above reflect the general time evolution of the PEI distributions and their components, and, therefore, do not show any changes that have occurred at a provincial level between the years in the study. Any change in the general distribution of a variable may be the consequence of multiple combinations of changes at a provincial level [98]. As a result, it is necessary to look at the annual mean variations distribution for each index. The mean values of the distributions (0.0074, for MYS and 0.0076, for EYS) as well as the standard deviations (0.0044, for MYS and 0.0041, for EYS) were virtually the same in both distributions. However, Fig 3 shows that the growth patterns differed by index, being more moderate in the case of MYS. There was also a group of provinces with a negative mean variation in the distribution of this component.

Despite there was an overall improvement in PEI values between 2006 and 2014, certain provinces which had a poorer educational development in 2006, such as Cañar or Bolívar and, particularly, Esmeraldas, were seen to be those which had improved most (annual mean variation [AMV] equal to 0.0099, 0.0108, and 0.0112, respectively), whereas others who exhibited greater initial educational development, such as El Oro or Guayas, had made less progress (respective AMV values equal to 0.0052 and 0.0041) (Fig 1). In this regard, our estimation of the Spearman’s rank correlation coefficient between the classification of Ecuadorian provinces as determined by the PEI in 2006 and the other defined by the AMV of the PEI was negative (-0.493). This fact indicated that the provinces which most increased their values between 2006 and 2014 were those that lagged furthest behind in 2006 with a lower PEI. This therefore pointed to a process of convergence in education amongst Ecuadorian provinces. Based on the provincial estimations of the education components of the PEI (S6 Table), the convergence between provinces was greater for the EYS index, since the absolute value of the Spearman’s rank correlation coefficient was closer to one (-0.734) and was extremely weak for the MYS index (-0.182) (Table 2).

**Table 2:** Provincial-level Education Index and its components: Spearman´s rank correlation coefficient between the index in 2006 and its annual mean variation (2006–2014).

	*ρ*	*p*
PEI	-0.493	0.011 *
MYS	-0.182	0.215
EYS	-0.734	0.000 **

Notes: PEI = Provincial-level Education Index. MYS = mean years of schooling index; EYS = expected years of schooling index: *ρ* = Spearman’s rank correlation coefficient; *p = p*-value from the one-tailed *t*-test;

* indicates statistical significance at the 5% level;

** indicates statistical significance at the 1% level.

Table 3 provides the CV^2^ values of the PEI and of its components for 2006 and 2014. These values give useful information on the degree of educational inequality among Ecuadorian provinces, particularly on its variation over time, as well as on the weight of each component in this inequality. Therefore, extending the previous results, we saw that the distributions of the PEI components reduced in inequality over the period studied. Nevertheless, the MYS distribution, which displayed the greatest level of inequality in the two years (CV^2^ equal to 0.0125, in 2006, and 0.0118, in 2014), was also the one to reduced said inequality less. In contrast, the CV^2^ values of the EYS in the two years showed that there was substantial homogeneity amongst Ecuadorian provinces in terms of this educational component, particularly in 2014, in which the CV^2^ presented a value close to zero. Nevertheless, the distribution of this indicator reduced its inequality across provinces between 2006 and 2014 by over 50%. In addition, overall educational inequality across provinces also fell between 2006 and 2014, according to the CV^2^ of the PEI in these years. As regards the contribution to overall inequality for the two years, the contribution of MYS was greater than that of EYS and was almost three times higher in 2014.

**Table 3:** Inequality in educational development and contribution of education indicators.

	2006			2014		
	MYS	EYS	PEI	MYS	EYS	PEI
CV^2^	0.013	0.002	0.004	0.012	0.001	0.003
% Contribution	63.18%	36.82%		77.31%	22.69%	

Notes: MYS = mean years of schooling index; EYS = expected years of schooling index: PEI = Provincial-level Education Index: CV^2^ = squared of the coefficient of variation.

To complement the information provided by the normalized education indices, it is worthwhile looking at the indicators behind them–mean years of schooling and expected years of schooling–, which allows us to understand the full meaning of the analysis. Table 4 shows the most common descriptive statistics in data analysis for these indicators. The mean value of the mean years of schooling indicator increases by slightly less than one year between 2006 and 2014 (0.885), while the increase is slightly more than one year for the mean of the expected years of schooling indicator (1.095).

**Table 4:** Descriptive summaries of education indicators.

	Mean years of schooling		Expected years of schooling	
	2006	2014	2006	2014
Mean	7.233	8.118	12.879	13.974
Standard deviation	0.809	0.881	0.642	0.480
Variation coefficient	0.112	0.108	0.050	0.034
Square of the variation coefficient	0.013	0.012	0.002	0.001
Maximum	9.285	10.248	14.284	14.768
Minimum	5.902	6.144	11.549	12.979
Range	3.383	4.104	2.735	1.789

Note: approximate results for the provinces of Amazonia in 2006 due to lack of representativeness in the survey.

From the statistical point of view, normalization implies a change of scale in the variable: a transformation with respect to which CV^2^ is invariant, as shown in the table. However, it is worth noting the values taken by the coefficient of variation (square root of CV^2^) for each of the indicators. Thus, for the mean years of schooling indicator, the dispersion represents 10.8% of its mean value in 2014, while, in the case of the expected years of schooling indicator, the variability represents only 3.4%.

Despite the reduction in educational inequality among Ecuadorian provinces, the range of the variable mean years of schooling in 2006 is around 3.4 years (from 5.902 in Bolivar to 9.285 in Pichincha), while, in 2014, this range reaches 4.1 years (from 6.144 in Chimborazo to 10.248 in Pichincha). However, when not considering Pichincha, the range is reduced to 3.1 years (from 6.144 in Chimborazo to 9.205 in Guayas). In the case of the expected years of schooling indicator, the range goes from 2.7 years in 2006 to 1.8 in 2014 (S5 Table).

**Fig 4:**
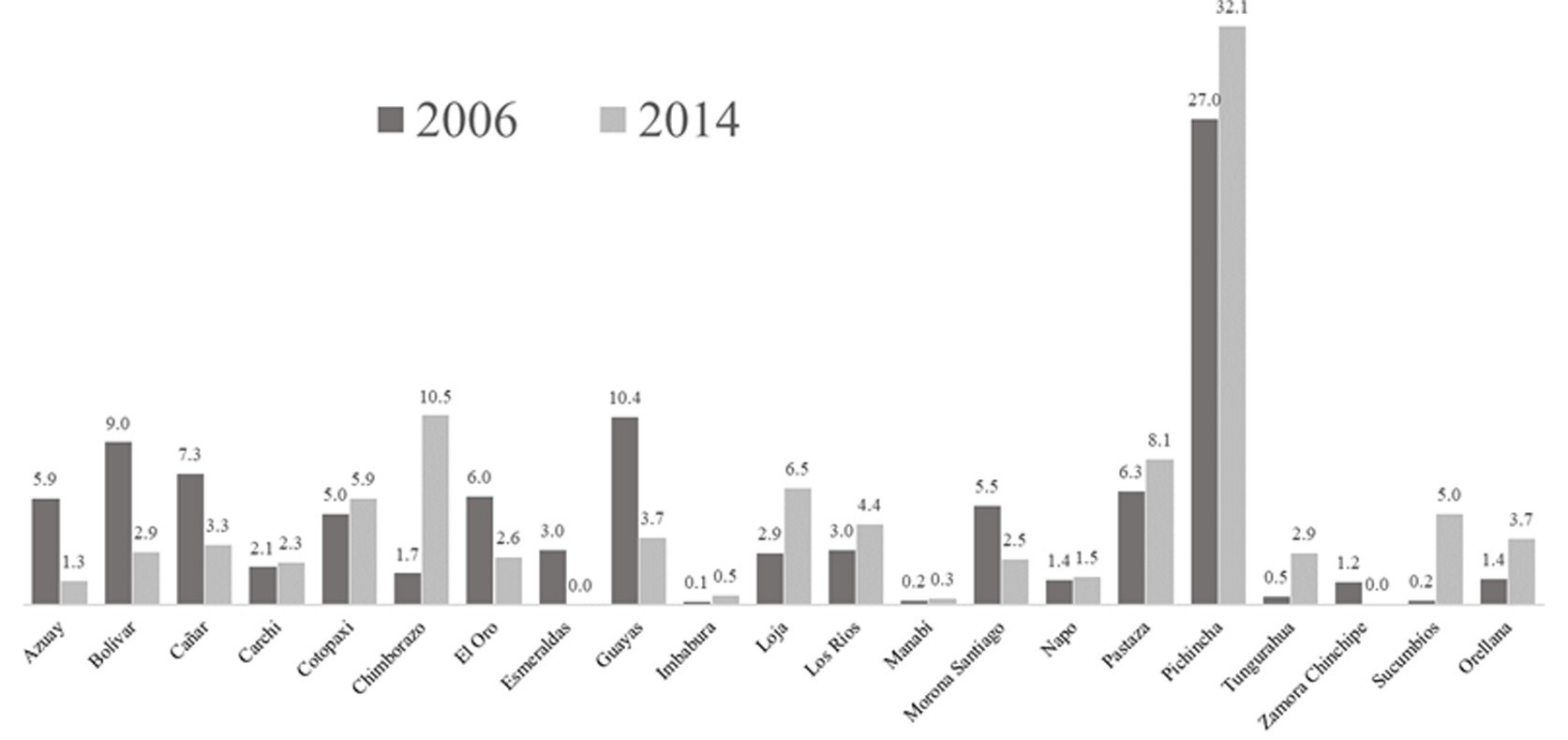
Provincial contribution to educational development overall inequality (%).

Data in Fig 4 show that the provinces with the greatest or lowest educational development drive inequality the most, whereas the lowest weight corresponded to provinces whose PEI values were closer to the mean value of the corresponding PEI distribution. Specifically, in 2006, the provinces of Pichincha and Guayas (with the highest PEI values) contributed most, in percentage terms, to overall educational inequality in Ecuador (27% and 10.4%, respectively), followed by Bolivar, with 9%, and Cañar, with 7.3% (provinces that evidenced a lower level of educational well-being in said year). In 2014, Pichincha (32.1%) and Chimborazo (10.5%) were the provinces with the highest and lowest PEI value, respectively, and which contributed most to educational inequality in Ecuador.

**Fig 5:**
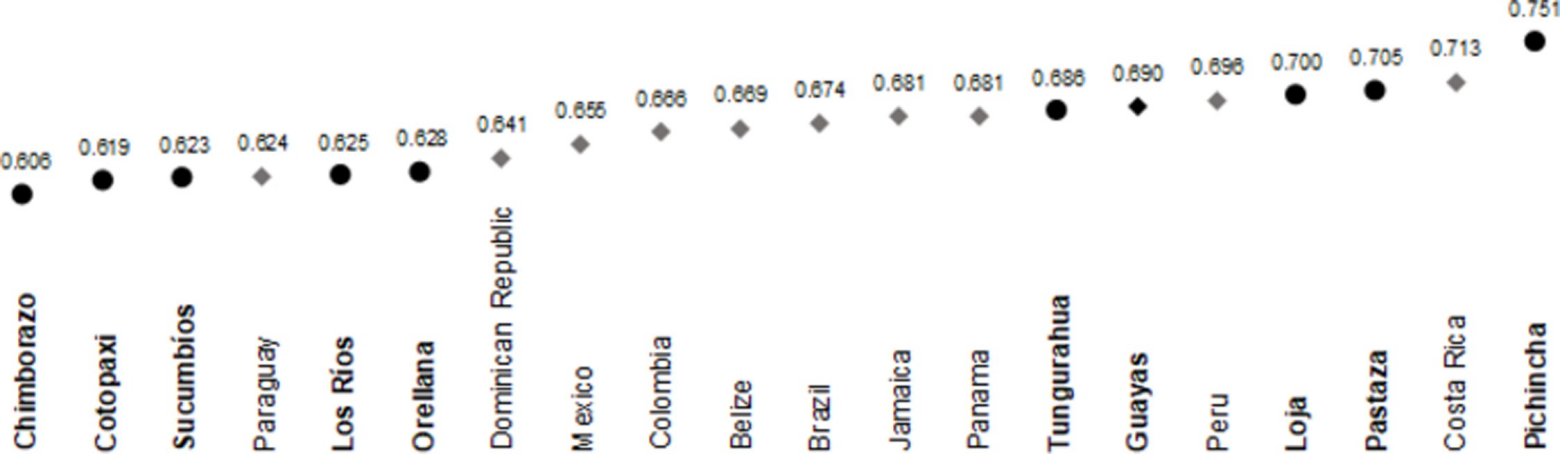
Comparison between the values of the Provincial-level Education Index of the provinces with the highest and lowest value of said index and the values of the education index of the Human Development Index for countries with an upper-middle income in Latin-America and the Caribbean. 2014. Note: Upper-middle income countries in 2014 are those with a gross national income per capita of between $4,126 and $12,735 according to the World Bank classification in 2014 [99].

Finally, Fig 5 shows the PEI values of those Ecuadorian provinces which had the best and worst educational development, together with the values of the educational component of the HDI of LAC countries that had an upper-middle income in 2014. As can be seen, the educational development of Ecuadorian provinces with a poorer PEI was lower than that of all the countries analyzed, except Paraguay, whose education index was lower than the PEI of Los Ríos and Orellana. Regarding the provinces with the highest PEI, Tungurahua and Guayas were only outperformed by Peru and Costa Rica, while only Costa Rica showed better results than Loja and Pastaza. It is worth highlighting the case of Pichincha, which displayed greater educational well-being than all the LAC countries considered.

## Discussion and conclusions

The Provincial-level Education Index shows an increase in the educational development of Ecuador’s provinces in the period under analysis. Yet despite this overall improvement, differences continue to exist. Thus, 12 of the 21 Ecuadorian provinces had a medium educational development in 2014 (PEI between 0.606 and 0.657), while the rest reached a high educational development (PEI between 0.677 and 0.751), according to the thresholds calculated from the distribution of the UNDP´s HDI educational index for that year. It is also worth mentioning that, with the exception of Pichincha, whose PEI was 0.751, the rest of the provinces were still far from 0.784, the threshold that determines the level of very high educational development.

In addition to Pichincha, the provinces with the highest PEI values in 2014 were Pastaza, Loja, Guayas, Tungurahua, and El Oro. At the other end of the scale were Chimborazo, Cotopaxi, Sucumbíos, and Los Ríos, which displayed the lowest values. Esmeraldas and Cañar witnessed the greatest increase in their PEI. When comparing the educational development of Ecuadorian provinces with that of LAC countries with the same socioeconomic characteristics (Paraguay, Dominican Republic, Mexico, Colombia, Belize, Brazil, Jamaica, Panama, Peru, and Costa Rica) it is worth noting that Tungurahua and Guayas were only surpassed by Peru and Costa Rica. Also, the cases of Pastaza and Loja must be highlighted, as they had a PEI only lower than that of Costa Rica. Finally, above all, Pichincha outstands for having a higher educational development than all the countries considered. All LAC countries analyzed presented better educational development than the provinces with the lowest PEI–with the exception of Paraguay, whose education index was lower than the PEI of Los Ríos and Orellana.

Our results are consistent with the data provided by official statistics regarding variables such as the literacy rate or the percentage of people in households with children who do not go to school. Thus, Pichincha presented the lowest rate of illiteracy in 2014, followed by Guayas and Loja (3.7%, 5.65%, and 5.8%, respectively), whereas the highest values corresponded to Chimborazo (19.4%) and Cotopaxi (13.7%), with Esmeraldas and Cañar evidencing the greatest reduction in illiteracy rates (from 13.4% to 8.5% and from 15.3% to 9.6%) during the study period [91]. In addition, Pichincha and Loja had the lowest percentage of people in households with children who did not go to school (0.5%), followed by Guayas (0.8%), while Chimborazo (1.1%) and Sucumbíos (1.6%) had the highest [91].

A wide range of reasons may lie behind our results. First, there is the need to consider the actual production structure and level of economic development of the various provinces. Amongst those evidencing the highest PEI values during the studied period, Pichincha, together with Guayas and Tungurahua, form part of the group of provinces displaying the greatest economic development [100]. These provinces have traditionally benefitted from better opportunities in terms of access to education and have also enjoyed a better-quality education system [101]. They also have the largest number of technical and technological institutes (over half of those in Ecuador are in Pichincha and Guayas), as well as the highest number of students enrolled at these institutions (over 65% between the two provinces) [102]. Bearing in mind the important role played by the oil sector in Ecuador’s provincial economic structure [100] and the economic boom that the country experienced during the study period, it comes as no surprise that certain oil provinces, such as Pastaza, evidenced some of the highest PEI values. This was mainly due to the favorable evolution of oil prices, which generated a significant amount public resources that were devoted to education, encouraging families to enroll their children in school. Sucumbíos, the most populated province in Amazonia, was amongst those to have recorded the lowest PEI values, even though it also exhibited a high degree of production specialization in the oil sector. This may be due to the existence of a dynamic labor market that significantly increases the opportunity costs of education, thereby leading to school lagging, dropout, and child labor. Finally, Chimborazo, one of the poorest provinces in the country (together with Bolívar, Orellana, and Sucumbíos) [100] showed the worst PEI value. With a high percentage of indigenous population, its economy is based on a subsistence model in which the agricultural sector, crafts and tourism are activities which tend to generate unskilled labor. Added to this is the strong rural component of the remaining provinces that had a worse PEI value (Cotopaxi and Los Ríos), which is exacerbated by the fact that the least qualified teachers tend to be concentrated in rural schools [48], and where there are also difficulties regarding access and inadequate school infrastructure (drinking water, electricity, and sanitation).

Other reasons behind our results might be due to the policies implemented by the country’s authorities at different administrative levels, the effects of which have begun to be felt during the study period. We are aware that no impact evaluation analyses have been carried out for most of them [103] and that therefore we cannot establish a cause-effect relation to determine what part of the observed effect might correspond to any given policy (net effect). However, said policies might account for the reduction in inequality in education, as already highlighted in some studies [28].

Among these policies, the Ten-Year Education Plan 2006–2015, which aimed to achieve quality basic education for all by 2015, is noteworthy. It also included the second of the Millennium Development Goals: achieving universal primary education by 2015 [42]. But, above all, mention should be made of the education reforms undertaken by the Ecuadorian government at all levels of education with the entry into force of the LOES of 2010 and the LOEI of 2011.

In addition, some free education policies were implemented with the aim of universalising basic education. These policies mainly affected decentralization in administrative, curricular, and financial decision making, and justified the coordination between the requirements of local development and what is offered by educational institutions, having economic consequences in the area [104]. This boosted parental participation in schools and led to an improvement in the way education is perceived by families [41]. In addition, the increase in the number of years of compulsory education at pre-school level helped to improve equity, as evidenced by our findings concerning the expecting years of schooling indicator. Various studies [105–108] show that early-childhood education exerts a positive effect on the likelihood of children being schooled afterwards, and in terms of preventing them from falling behind or dropping out. Early-childhood education also seems to positively impact learning outcomes in the early years of primary education, with these effects proving to be greater in the case of children from poor families. Expanding access to early-childhood education is key to enhancing the effectiveness of the education system and curbing inequality [56], as the literature has evidenced the positive effects that early-childhood education has on disadvantaged children [109].

Secondly, there is the need to consider a series of fee-free policies in education implemented during the period covered in this research, and which might have led to improvements in the component related to expected years of schooling, the estimation of which involves attendance and enrolment rates.

Prominent among these policies are, on the one hand, the waiving of school fees at all educational levels, which was gradually implemented from 2007 onwards. And, on the other hand, the provision of free schoolbooks, which initially covered the first to the tenth year of General Basic Education, and which was later extended to all levels of non-schooled education [110, 111]. Added to it is the program of free schoolbooks in ancestral languages *Kukayus Pedagógicos*, which has been developed mainly in the provinces of the Sierra region (Pichincha, Cotopaxi, Imbabura, Tungurahua, Bolívar, Chimborazo, Cañar, Azuay, and Loja), where a large part of the country’s indigenous population lives. Also, the *Programa de Alimentación Escolar*, which begun in 1999, must be highlighted, as it provides breakfast and lunch for children in vulnerable households, offering two meals a day in schools–only one after 2007– for students of Initial Education and General Basic Education in urban and rural areas. All provinces improved their coverage over the period, both in the textbook and school meals programs, and the provinces that initially displayed a lower PEI (Chimborazo and Cotopaxi in the former, and Azuay and Los Ríos, in the latter, respectively) (S7 Table) showed the greatest increase. Mention should be made of the *Hilando el Desarrollo* program, through which the uniforms of students in state schools at the levels of Initial Education and General Basic Education are paid. This program promotes school enrolment, since, as uniforms are compulsory in Ecuador, not having one might pose a hurdle to attending school and urge children to drop out. The program, which begun in 2007 and which is still in place today, also promotes associations of small craftsmen and women in micro-textile firms to make uniforms in the local area by granting microcredits, training, and technical assistance. Guayas and Pichincha, two of the provinces with the highest PEI values, evidence the greatest reduction in the percentage of students benefiting from free uniforms between 2009 and 2012 (-11.1 and -10.8, respectively) (S7 Table). It is worth noting that the results of an assessment of the program’s impact in five provinces of the Costa region indicated that the program had no effect on school enrolment, although it did show negative effects *vis-à-vis* attendance [112]. These results could be due to the sunk costs fallacy and to parental disappointment with schools, which, after promising a uniform, failed to supply one. Other aspects that influenced the results were the poor quality of the uniforms provided by the program, and the stigmatization suffered by the children who wore it [112]. Overall, the results obtained might be shedding light on certain progress in terms of improvements in interprovincial educational equality, although differences can still be observed.

Although the aforementioned policies correspond to the central government and are implemented at the national level, they could be behind our results–especially those related to certain provinces that have shown a better performance in the PEI between the two periods studied−. Although all these policies have been an important incentive for the access and continuity of children in the school system throughout the country, the greatest effort has been made in rural areas [113]. As a result, some of the provinces with the highest proportion of rural population have also been some of the ones that have shown the best evolution in their PEI. In addition, it should be noted that most of the provincial governments of Costa and Sierra continued to allocate their own budget to some of these programs −such as free textbooks−, which made it possible to reach a greater number of students [114] (S7 Table). Institutions play a key role in the way societies distribute the benefits and costs of public policies implemented at different levels of government. Without them, there is little likelihood that the necessary change will take place. The fact that some institutions work better than others at the regional/local level may cause a national program/policy to obtain better results in some provinces than in others (inclusive institutions vs. extractive institutions) [115].

Together with these central government policies implemented at a national scale, some subnational governments also developed various initiatives. Such is the case of Pichincha which, between 2004 and 2007, introduced a program of free schoolbooks: *Sí a la Educación Básica (Sílabas)*. Since 2006, this program has also been in place in the provinces of Azuay and Manabí. In Guayas, the *Más Libros* program is still being implemented today [110, 116].

Finally, the effects of the *Bono de Desarrollo Humano* (BDH) must also be considered. Most of the studies which assess the impact of CCT programs in LAC countries report significant effects in the increase of school enrolment and in the reduction in child labor. This is the case of some research conducted in countries with upper-middle income in the region, the results of which reflect the positive effects that these programs have on school attendance and enrolment, as occurred in *Oportunidades* (formerly *Progresa*) in Mexico [117, 118], *Familias en Acción* in Colombia [119] or *Bolsa Escola* in Brazil [120]. In Ecuador, BDH’s achievements have been highlighted by studies that find significant effects in the reduction of activity and child labor rates, delaying children’s entry into the labor force [121]. This conclusion is supported by the fact that such transfers considerably increase school enrollment, and that this effect is four times greater in households that perceive this conditionality as an obligation compared to those who feel that the transfer is not conditional [122].

During 2009–2014, the BDH had greater coverage in some of the provinces where the education index shows a clear improvement −such as Esmeraldas, Loja, Morona Santiago, Tungurahua, and Zamora Chinchipe−. The beneficiaries’ fear of being sanctioned in the event of a verification of conditionality in education has probably contributed to the results achieved [123].

Finally, it is worth mentioning that, even though no cause-effect relation may be inferred from our study, the convergence in educational development of Ecuadorian provinces is consistent with studies that point to a process of income convergence at a provincial level in Ecuador in the period covered in this research, and which underpin the importance of reducing inequalities in education in order to achieve such a convergence [45,124, 125].

In spite of this, the convergence in education obtained should be considered with the caution that the lack of representativeness of the Amazonian provinces in the fifth round of the LSMS implies. In line with certain authors who consider that a sample can be valid without being representative [126, 127] our analysis of the causes behind the results obtained will bring us closer to the scientific objective of understanding the territorial dynamics of educational development in the Ecuadorian provinces. In any case, the main limitation in our work is the lack of data. The fact that the latest available round of the LSMS corresponds to the period 2013–2014 does not −for the time being− allow us to analyze the consequences of Ecuador’s educational policies with a greater perspective, particularly if we consider that the outcomes of educational policies do not tend to emerge in the short term. In a similar vein, the LSMS’s lack of representativeness for a higher level of disaggregation (Ecuadorian cantons) prevented us from delving more deeply into the dynamics of educational inequalities in Ecuador. We are also aware that using only two indicators is perhaps a reductionist way of understanding a country’s educational development. Nevertheless, the fact that they are the indicators used by the UNDP allows our technique to be used for drawing comparisons, such as those made in this work between the educational development of the Ecuadorian provinces and that of the upper-middle income LAC countries. It should also be noted that the use of the mean years of schooling variable in the comparison of educational development between countries has been criticized by several authors [9, 128]. The reason lies in the errors that can occur in the interpretation of the results due to the existing heterogeneity between countries’ educational systems. However, this criticism does not make sense in this work, since the PEI is calculated for provinces that have the same educational system. Finally, although the LSMS did allow for the estimation of the education indicators used in this research, the results must be analyzed with the due caution required for a study that is based on the use of a survey.

If no new rounds of the LSMS were to be conducted, an alternative to continue analyzing the evolution of the country’s educational development would be to use an employment survey, such as the Ecuador Employment, Unemployment and Underemployment Survey. Although these types of household surveys focus on labor issues and are less ambitious than the LSMS, they also include demographic and socioeconomic aspects of the surveyed population [75].

Furthermore, considering that each generation grows up with higher levels of education than past generations [129], it would be interesting to analyze the effect of age on educational development in Ecuadorian provinces. Since it is to be expected that the provinces with the highest mean age are also those with the lowest mean years of schooling, the estimation of an Educational Index at the provincial level by age groups–following the procedure described in this paper–could be a future line of research. This estimate could complement the information on the territorial pattern of education in Ecuador provided in this paper.

In addition, the design of educational indices based on other educational variables, such as enrollment rates at different educational levels, could also be an interesting complement to the Provincial-level Education Index. Finally, new indexes could be designed to show educational inequality between subpopulations determined by characteristics such as geographic location (rural vs. urban) or gender. This would allow a deeper understanding of Ecuador’s sociodemographic reality and its horizontal inequalities.

## Conclusions

Using the latest micro-databases available for the LSMS, we estimated a Provincial-level Education Index which showed a favorable evolution in educational development in Ecuadorian provinces as well as a slight reduction in inequality amongst them. However, despite this general improvement, there are still differences at the provincial level.

Our findings are consistent with the production structure and socioeconomic features of Ecuadorian provinces and evidence the effects that certain public policies might have had in the field of education. Nevertheless, the country’s efforts in terms of implementing policies aimed at reducing poverty (National Plans for Good Living and the BDH), on the one hand, and those which seek to improve human capital and its capabilities (Ten-Year Education Plan 2006–2015, and the latest educational reforms), on the other, might not have proved sufficient to bring Ecuador out of the middle-income trap [70] in which it has been immersed for over 60 years [130]. In other words, Ecuador is caught in a low productivity growth, which keeps the country trapped between low income and high income due to structural problems that are hard to overcome. These problems prevent the country from boosting its level of development in general, and its level of educational development, in particular. Although it is true that, during the boom in oil prices (2007–2014), Ecuador experienced a period of economic growth which triggered a reduction in poverty and a redistribution of educational opportunities, certain structural problems still remain. Although investment in education increased during the years of the study, rising from 2.3% of GDP in 2006 to 4.7% in 2014 [91], the country must still address various issues. Firstly, a greater investment in school infrastructure is necessary, given that both the difficulties in accessing educational services and their quality partly account for differences between urban, rural, and indigenous areas. These differences only heighten the risk of poverty and social exclusion in certain provinces in the country. Secondly, continuous teacher training should be improved in order to enable teachers to successfully respond to society’s new demands. Also, teacher mobility between urban and rural areas should be fostered [131], and, finally, external standardized tests to measure students’ learning outcomes and academic performance that would provide guidelines to improve curricula should be applied [41]. A further issue that requires action is the high degree of segregation (urban-rural and ethnic income), which is mainly observed in General Basic Education, and which evidences major differences between provinces. All of this would help to build a fairer education system and to expand equal opportunities [132]. These are just some of the challenges that Ecuador’s education policies must face in the coming decades.

In order to gain insight into the distribution of a country’s well-being, it is necessary to analyze the distribution of its educational outcomes, as well as their evolution over time. In LAC, a region with large gaps in access to education, the analysis of educational attainment is particularly important [133]. Education data provided by LSMSs offer valuable information for authorities to implement public policies aimed at improving citizens’ living conditions [47]. However, data are worthless if their interpretation is not based on knowledge of the underlying reality. Thus, as this research has shown for Ecuador, understanding the educational situation behind the microdata provided by a country’s household survey and, in particular, by a LSMS, allows for the definition of educational development indices at the national and subnational levels. The values of these indices could provide a guide for the design and implementation of policies to ensure coverage of educational demand and excellence in education, as well as the equality inherent in the right to education for all.

## Supporting information

S1 Table Structure of the National Education System (non-university education) in Ecuador affecting the fifth round 2005–2006 and sixth round 2013–2014 of the Ecuadorian Living Standards Measurement Survey. (DOCX)

S2 Table Structure of the National Education System (non-university education) in Ecuador affecting the sixth round 2013–2014 of the Ecuadorian Living Standards Measurement Survey. (DOCX)

S3 Table Procedure for estimating the mean years of schooling indicator in the fifth round of the LSMS (2005–2006). (DOCX)

S4 Table Procedure for estimating the mean years of schooling indicator in the sixth round of the LSMS (2013–2014). (DOCX)

S5 Table Values of the mean years of schooling and expected years of schooling indicators. 2006 and 2014.
(DOCX)

S6 Table Values of the provincial-level education index and of its components, and annual average variation. 2006 and 2014.
(DOCX)

S7 Table Percentage of students benefiting from free schoolbooks, school meals and uniforms. (DOCX)
