# Supplementary material for: Education as a dimension of human development: A Provincial-level Education Index for Ecuador
Source: PLoS One. 2022 Jul 8;17(7):e0270932. doi: 10.1371/journal.pone.0270932 (PMC9269385; doi:10.1371/journal.pone.0270932)
Supplement: S1 Table — (DOCX) [file pone.0270932.s001.docx]

**S1 Table. Structure of the National Education System (non-university education) in Ecuador affecting the fifth round 2005-2006 and sixth round 2013-2014 of the Ecuadorian Living Standards Measurement Survey**

| Level | Sublevel | Number of years of study | Official age  (years) |
| --- | --- | --- | --- |
| Pre-primary | Kindergarten | 1 | 4 to 5 |
|  |  | 1 | 5 to 6 |
| Primary | First cycle | 2 | 6 and 7 |
|  | Second cycle | 2 | 8 and 9 |
|  | Third cycle | 2 | 10 and 11 |
| Middle school | Basic | 3 | 12 to 14 |
|  | Diversified, comprising:   - Short post-basic cycle courses - Baccalaureate | 3  1 or 2  3 | 15 to 17 |
|  | Specialisation, Post-baccalaureate | 2 | 18 and 19 |
|  | Arts and Crafts | Subject to regulation by the education authority |  |
