# Supplementary material for: Education as a dimension of human development: A Provincial-level Education Index for Ecuador
Source: PLoS One. 2022 Jul 8;17(7):e0270932. doi: 10.1371/journal.pone.0270932 (PMC9269385; doi:10.1371/journal.pone.0270932)
Supplement: S2 Table — (DOCX) [file pone.0270932.s002.docx]

**S2 Table. Structure of the National Education System (non-university education) in Ecuador affecting the sixth round 2013-2014 of the Ecuadorian Living Standards Measurement Survey**

| Level | Sublevel | Number of years of study | Official age  (years) |
| --- | --- | --- | --- |
| Initial Education | Initial 1 (non-schooled) | - | Under 3 |
|  | Initial 2 (schooled) | 3 | 3 to 5 |
| General Basic Education | Basic Preparatory | 1 | 5 |
|  | Basic Elemental | 3 | 6 to 8 |
|  | Basic Middle | 3 | 9 to 11 |
|  | Basic Higher | 3 | 12 to 14 |
| Baccalaureate | General Unified Baccalaureate in Sciences or Technical Disciplines (1st, 2nd, 3rd) | 3 | 15 to 17 |
| Complementary Baccalaureate | Technical Production ^a^ | 1 | Over 17 |
|  | Arts and Crafts | Subject to regulation by the  education authority |  |

Notes: ^a^ Complementing de Technical Baccalaureate (optional).
