# Supplementary material for: Education as a dimension of human development: A Provincial-level Education Index for Ecuador
Source: PLoS One. 2022 Jul 8;17(7):e0270932. doi: 10.1371/journal.pone.0270932 (PMC9269385; doi:10.1371/journal.pone.0270932)
Supplement: S3 Table — (DOCX) [file pone.0270932.s003.docx]

**S3 Table. Procedure for estimating the mean years of schooling indicator in the fifth round of the LSMS (2005-2006)**

| Question Pe45:  Highest level of education attended or completed | Question Pe46:  Level or highest year of education passed | Maximum number of years passed ^a^ | Remarks |
| --- | --- | --- | --- |
| Primary | Number of years of the answer | 6 |  |
| Basic Education | Number of years of the answer | 9 | At the time of applying the fifth round of the LSMS, none of those interviewed could have taken basic education since the age of five. As a result, the maximum number of years (9) would correspond to six years of the primary cycle plus the three years of the basic compulsory and common basic middle level. |
| Middle Education or Baccalaureate | Primary + Number of years of the answer | 12 | One of the education levels of Pe45 question is “Secondary”. Although there is no education level corresponding to this name in the legislation, “Middle Education or Baccalaureate” was taken as an equivalent level.  It should be remembered that the “Middle Education” level is made up of the basic, diversified and specialisation (post-Baccalaureate) cycles. The survey considers the level “post-Baccalaureate” to be an independent level such that, as regards Pe45, the “Middle Education” level only comprises its first two cycles. |
| Post-Baccalaureate/  Non-university Higher Education | Primary + Middle Education + Number of years of the answer | 15 | This considers the qualification of post-Baccalaureate with two years of study, in accordance with Article 86c) of the General Rules of the Education Act (1985). In the case of the Post-Baccalaureate Specialisation cycle, for training technicians and technologists, taught at the Higher Technical and Technological Institutes, training may last two or three years, in accordance with Article 163 of the General Rules of the Education Act (1985). |
| Higher Education | Primary + Middle Education + Number of years of the answer | 17 | The degree in Human Medicine lasts six years, such that the maximum number of years passed would be 18. For interviewees holding post-Baccalaureate qualifications in question Pe47 (what was the last document you obtained?) the maximum could be 19 years. |
| Postgraduate | Primary + Middle Education + Higher Education + Number of years of the answer | 19 | Given the vast number of postgraduate studies and the varying durations of each, we opted to consider the years stated by the interviewee in their answer in those cases where the duration of the postgraduate course exceeded two years. |

Note: ^a^ This considers the years passed since the first year of compulsory schooling.
