# Supplementary material for: Education as a dimension of human development: A Provincial-level Education Index for Ecuador
Source: PLoS One. 2022 Jul 8;17(7):e0270932. doi: 10.1371/journal.pone.0270932 (PMC9269385; doi:10.1371/journal.pone.0270932)
Supplement: S4 Table — (DOCX) [file pone.0270932.s004.docx]

**S4 Table. Procedure for estimating the mean years of schooling indicator in the sixth round of the LSMS (2013-2014)**

| Question Pe47:  Highest level of education passed | Question 48: Number of years passed | Maximum number of years passed ^a^ | Remarks |
| --- | --- | --- | --- |
| Primary | Number of years of the answer | 6 |  |
| Basic Education | Number of years of the answer | 10 |  |
| Middle Education or Baccalaureate | Primary + Number of years of the answer  or  Basic Education + Number of years of the answer | 13 | One of the education levels of the Pe47 question is “Secondary”. Although there is no education level corresponding to this name in the legislation, “Middle Education or Baccalaureate” was taken as an equivalent level. It should be remembered that the “Middle Education” level is made up of the basic, diversified and specialisation (post-Baccalaureate) cycles. The survey considers the level “post-Baccalaureate” to be an independent level such that, as regards Pe47, the “Middle Education” level only comprises its first two cycles. |
| Post-Baccalaureate/  Non-university Higher Education | Primary + Middle Education + Number of years of the answer  or  Basic Education + Baccalaureate + Number of years of the answer | 15 | This considers the qualification of post-Baccalaureate with two years of study, in accordance with Article 86c) of the General Rules of the Education Act (1985). In the case of the post-Baccalaureate Specialisation cycle, for training technicians and technologists, taught at the Higher Technical and Technological Institutes, training may last two or three years, in accordance with Article 163 of the General Rules of the Education Act (1985). |
| Higher Education | Primary + Middle Education + Number of years of the answer  or  Basic Education + Baccalaureate + Number of years of the answer | 18 | The degree in Human Medicine lasts six years, such that the maximum number of years passed would be 19.  Curricular reform for Basic Education introduced this level as of 1996. As a result, although it is not possible for an interviewee to have completed their studies starting Basic Education from the first year, it is possible for those who started Primary Education, and who at some point during their education have accredited their training with the Basic Education diploma. |
| Postgraduate | Primary + Middle Education + Superior + Number of years of the answer  or  Basic Education + Baccalaureate + Higher Education + Number of years of the answer | 20 | As in the case above, although it is not possible for an interviewee to have completed their studies starting Basic Education from the first year, it is possible for those who started Primary Education, and who at some point during their education have accredited their training with the Basic Education diploma. |

Notes: ^a^ This considers the years passed since the first year of compulsory schooling.
