# Supplementary material for: Education as a dimension of human development: A Provincial-level Education Index for Ecuador
Source: PLoS One. 2022 Jul 8;17(7):e0270932. doi: 10.1371/journal.pone.0270932 (PMC9269385; doi:10.1371/journal.pone.0270932)
Supplement: S5 Table — 2006 and 2014. (DOCX) [file pone.0270932.s005.docx]

**S5 Table. Values of the mean years of schooling and expected years of schooling indicators. 2006 and 2014**

| Province | Mean years of schooling | | | Expected years of schooling | | |
| --- | --- | --- | --- | --- | --- | --- |
|  | 2006 | 2014 | | 2006 | | 2014 |
| Azuay | 8.056 | | 8.462 | 13.467 | 14.228 | |
| Bolívar | 5.902 | | 7.294 | 12.525 | 13.965 | |
| Cañar | 5.913 | | 7.450 | 12.711 | 13.715 | |
| Carchi | 6.857 | | 7.398 | 12.394 | 13.948 | |
| Cotopaxi | 6.258 | | 6.907 | 12.593 | 14.014 | |
| Chimborazo | 6.888 | | 6.144 | 12.437 | 14.452 | |
| El Oro | 8.126 | | 8.901 | 13.396 | 13.975 | |
| Esmeraldas | 7.203 | | 8.184 | 11.788 | 13.837 | |
| Guayas | 8.538 | | 9.205 | 13.409 | 13.789 | |
| Imbabura | 7.416 | | 7.974 | 12.899 | 14.558 | |
| Loja | 7.797 | | 8.693 | 13.299 | 14.768 | |
| Los Ríos | 7.055 | | 7.574 | 11.965 | 13.397 | |
| Manabí | 7.054 | | 7.727 | 12.795 | 14.126 | |
| Morona Santiago | 7.071 | | 7.976 | 11.549 | 13.224 | |
| Napo | 7.696 | | 8.785 | 13.083 | 13.898 | |
| Pastaza | 7.422 | | 9.198 | 14.284 | 14.337 | |
| Pichincha | 9.285 | | 10.248 | 13.791 | 14.726 | |
| Tungurahua | 7.196 | | 8.556 | 13.363 | 14.438 | |
| Zamora Chinchipe | 6.731 | | 8.100 | 12.759 | 13.893 | |
| Sucumbíos | 6.984 | | 7.687 | 12.891 | 13.187 | |
| Orellana | 6.440 | | 8.015 | 13.059 | 12.979 | |

Source: authors’ calculations based on the micro databases of the fifth round and sixth round of the *Encuesta de Condiciones de Vida* (INEC 2007b, 2014a).
