# Supplementary material for: Education as a dimension of human development: A Provincial-level Education Index for Ecuador
Source: PLoS One. 2022 Jul 8;17(7):e0270932. doi: 10.1371/journal.pone.0270932 (PMC9269385; doi:10.1371/journal.pone.0270932)
Supplement: S6 Table — 2006 and 2014. (DOCX) [file pone.0270932.s006.docx]

**S6 Table. Values of the provincial-level education index and of its components, and annual average variation. 2006 and 2014**

| Province | 2006 | | |  | 2014 | | |  | AMV | | |
| --- | --- | --- | --- | --- | --- | --- | --- | --- | --- | --- | --- |
|  | MYS | EYS | PEI |  | MYS | EYS | PEI |  | MYS | EYS | PEI |
| Azuay | 0.537 | 0.748 | 0.643 |  | 0.564 | 0.790 | 0.677 |  | 0.0034 | 0.0053 | 0.0043 |
| Bolívar | 0.393 | 0.696 | 0.545 |  | 0.486 | 0.776 | 0.631 |  | 0.0116 | 0.0100 | 0.0108 |
| Cañar | 0.394 | 0.706 | 0.550 |  | 0.497 | 0.762 | 0.629 |  | 0.0128 | 0.0070 | 0.0099 |
| Carchi | 0.457 | 0.689 | 0.573 |  | 0.493 | 0.775 | 0.634 |  | 0.0045 | 0.0108 | 0.0076 |
| Cotopaxi | 0.417 | 0.700 | 0.558 |  | 0.460 | 0.779 | 0.619 |  | 0.0054 | 0.0099 | 0.0076 |
| Chimborazo | 0.459 | 0.691 | 0.575 |  | 0.410 | 0.803 | 0.606 |  | -0.0062 | 0.0140 | 0.0039 |
| El Oro | 0.542 | 0.744 | 0.643 |  | 0.593 | 0.776 | 0.685 |  | 0.0065 | 0.0040 | 0.0052 |
| Esmeraldas | 0.480 | 0.655 | 0.568 |  | 0.546 | 0.769 | 0.657 |  | 0.0082 | 0.0142 | 0.0112 |
| Guayas | 0.569 | 0.745 | 0.657 |  | 0.614 | 0.766 | 0.690 |  | 0.0056 | 0.0026 | 0.0041 |
| Imbabura | 0.494 | 0.717 | 0.605 |  | 0.532 | 0.809 | 0.670 |  | 0.0047 | 0.0115 | 0.0081 |
| Loja | 0.520 | 0.739 | 0.629 |  | 0.580 | 0.820 | 0.700 |  | 0.0075 | 0.0102 | 0.0088 |
| Los Ríos | 0.470 | 0.665 | 0.568 |  | 0.505 | 0.744 | 0.625 |  | 0.0043 | 0.0099 | 0.0071 |
| Manabí | 0.470 | 0.711 | 0.591 |  | 0.515 | 0.785 | 0.650 |  | 0.0056 | 0.0092 | 0.0074 |
| Morona Santiago | 0.471 | 0.642 | 0.556 |  | 0.532 | 0.735 | 0.633 |  | 0.0075 | 0.0116 | 0.0096 |
| Napo | 0.513 | 0.727 | 0.620 |  | 0.586 | 0.772 | 0.679 |  | 0.0091 | 0.0057 | 0.0074 |
| Pastaza | 0.495 | 0.794 | 0.644 |  | 0.613 | 0.796 | 0.705 |  | 0.0148 | 0.0004 | 0.0076 |
| Pichincha | 0.619 | 0.766 | 0.693 |  | 0.683 | 0.818 | 0.751 |  | 0.0080 | 0.0065 | 0.0073 |
| Tungurahua | 0.480 | 0.742 | 0.611 |  | 0.570 | 0.802 | 0.686 |  | 0.0113 | 0.0075 | 0.0094 |
| Zamora Chinchipe | 0.449 | 0.709 | 0.579 |  | 0.540 | 0.772 | 0.656 |  | 0.0114 | 0.0079 | 0.0096 |
| Sucumbíos | 0.466 | 0.716 | 0.591 |  | 0.512 | 0.733 | 0.623 |  | 0.0059 | 0.0021 | 0.0040 |
| Orellana | 0.429 | 0.726 | 0.577 |  | 0.534 | 0.721 | 0.628 |  | 0.0131 | -0.0006 | 0.0063 |

Notes: MYS = mean years of schooling index; EYS = expected years of schooling index; PEI = Provincial-level Education Index; AMV = annual mean variation.

Source: authors’ calculations based on the micro databases of the fifth round and sixth round of the *Encuesta de Condiciones de Vida* (INEC 2007b, 2014a).
