# Supplementary material for: Education as a dimension of human development: A Provincial-level Education Index for Ecuador
Source: PLoS One. 2022 Jul 8;17(7):e0270932. doi: 10.1371/journal.pone.0270932 (PMC9269385; doi:10.1371/journal.pone.0270932)
Supplement: S7 Table — (DOCX) [file pone.0270932.s007.docx]

**S7 Table.** **Percentage of students benefiting from free schoolbooks, school meals and uniforms.**

| Province | School books ^a^ | | School meals ^b^ | | Uniforms ^c^ | |
| --- | --- | --- | --- | --- | --- | --- |
|  | 2006 | 2013 | 2007 | 2013 | 2009 | 2012 |
| Azuay | 54.1 | 94.8 | 39.0 | 80.8 | 44.0 | 47.5 |
| Bolívar | 61.5 | 95.3 | 63.9 | 86.2 | 64.0 | 67.2 |
| Cañar | 56.9 | 96.1 | 50.5 | 82.0 | 50.4 | 54.3 |
| Carchi | 64.7 | 95.5 | 64.0 | 80.4 | 37.3 | 47.6 |
| Chimborazo | 52.9 | 94.6 | 69.8 | 89.2 | 50.6 | 57.1 |
| Cotopaxi | 56.1 | 94.2 | 59.4 | 68.7 | 67.1 | 67.9 |
| El Oro | 65.0 | 89.8 | 38.3 | 59.5 | 35.9 | 29.8 |
| Esmeraldas | 66.6 | 92.9 | 77.5 | 78.0 | 52.1 | 53.8 |
| Guayas | 65.8 | 92.4 | 50.1 | 55.1 | 36.1 | 25.0 |
| Imbabura | 56.8 | 93.0 | 62.8 | 76.5 | 44.2 | 48.8 |
| Loja | 60.2 | 96.3 | 63.1 | 80.1 | 59.5 | 53.3 |
| Los Ríos | 66.7 | 94.8 | 43.5 | 76.8 | 45.6 | 48.3 |
| Manabí | 69.0 | 93.2 | 64.2 | 80.0 | 59.5 | 49.4 |
| Pichincha | 49.2 | 88.6 | 32.0 | 60.0 | 36.3 | 25.5 |
| Tungurahua | 46.1 | 91.1 | 54.5 | 85.3 | 47.1 | 49.2 |

Notes: ^a^ Percentage of students from 5 to 14 years old who attend public education schools that receive free school books out of the total number of children aged 5 to 14 who attend this type of school during the period; ^b^ Percentage of students enrolled in primary education (5 to 11 years old) who receive breakfast at school during the period out of the total number of children enrolled in primary education (5 to 11 years old); ^c^ Percentage of students (5 to 11 years old) who attend public education schools who receive free school uniforms out of the total number of children aged 5 to 11 who attend this type of school.

Source: authors’ own based on the *Sistema Integrado de Conocimiento y Estadística Social* de Ecuador (*Secretaría Nacional de Planificación y Desarrollo* 2019). At: <http://www.conocimientosocial.gob.ec/home.jsf>.
